# Single-use bronchoscopes for pleural cavity access: cadaveric feasibility study

**DOI:** 10.1007/s11748-026-02309-4

**Published:** 2026-05-11

**Authors:** Hiroki Imabayashi, Yuki Sata, Terunaga Inage, Kazuhisa Tanaka, Takane Suzuki, Hidemi Suzuki

**Affiliations:** 1 Department of General Thoracic Surgery, Chiba University Graduate School of Medicine, 1-8-1 Inohana, Chuo-ku, Chiba, 260-8670 Japan; 2 Department of Bioenvironmental Medicine, Chiba University Graduate School of Medicine, 1-8-1 Inohana, Chuo-ku, Chiba, 260-8670 Japan

**Keywords:** Single-use bronchoscope, Pleuroscopy, Thoracoscopy, Chest drain, Cadaver

## Abstract

We conducted a cadaveric feasibility study of two intrapleural applications of single-use bronchoscopes. In Approach 1, a 2.7 mm bronchoscope was used as a visual stylet inside a 20-Fr trocar catheter to confirm pleural entry and drain-tip position. In Approach 2, a 5.0 mm bronchoscope was used as a flexible thoracoscope for limited pleural inspection, aspiration, and direct-vision forceps biopsy; a sprayed-instillation maneuver was performed only to confirm technical deliverability. Approach 1 was attempted and feasible in six hemithoraces of three Thiel-embalmed cadavers. Approach 2 was attempted and feasible in four hemithoraces; it was not attempted in two hemithoraces due to time constraints and a large persistent air leak after Approach (1) No negative-to-positive conversion (new persistent air leak) was observed after Approach (2) These techniques may offer complementary approaches to conventional methods for observing the intrathoracic space and therefore warrant stepwise clinical validation.

**Supplementary Information:**

The online version contains supplementary material available at 10.1007/s11748-026-02309-4.

## Introduction

Semi-rigid pleuroscopy provides thoracoscopic diagnosis with results comparable to those of rigid thoracoscopy; however, it requires dedicated equipment [1, 2]. In the absence of such equipment, pleural inspection and biopsy have been performed using a flexible bronchoscope inserted via a chest-tube tract or sheath-based access [3, 4]. Moreover, a retrospective comparison suggested similar diagnostic yield and complication profiles between flexible bronchoscope-based medical thoracoscopy and semi-rigid pleuroscopy [5].

We describe two technique-focused intrapleural applications of single-use bronchoscopes for thoracic surgeons and interventional pulmonologists: a 2.7 mm scope as a visual stylet for direct-vision drain entry or tip confirmation (Approach 1) and a 5.0 mm scope as a flexible thoracoscope for limited pleural inspection or sampling (Approach 2). These adjuncts may help with rapid bedside drain confirmation or limited diagnostic pleural workup when pleuroscopy or Video-Assisted Thoracoscopic Surgery (VATS) is not readily available; however, standard pleuroscopy or VATS should be preferred when feasible. Herein, we report the technical feasibility and workflow of using single-use bronchoscopes.

## Specimens and setup

**Fig. 1:**
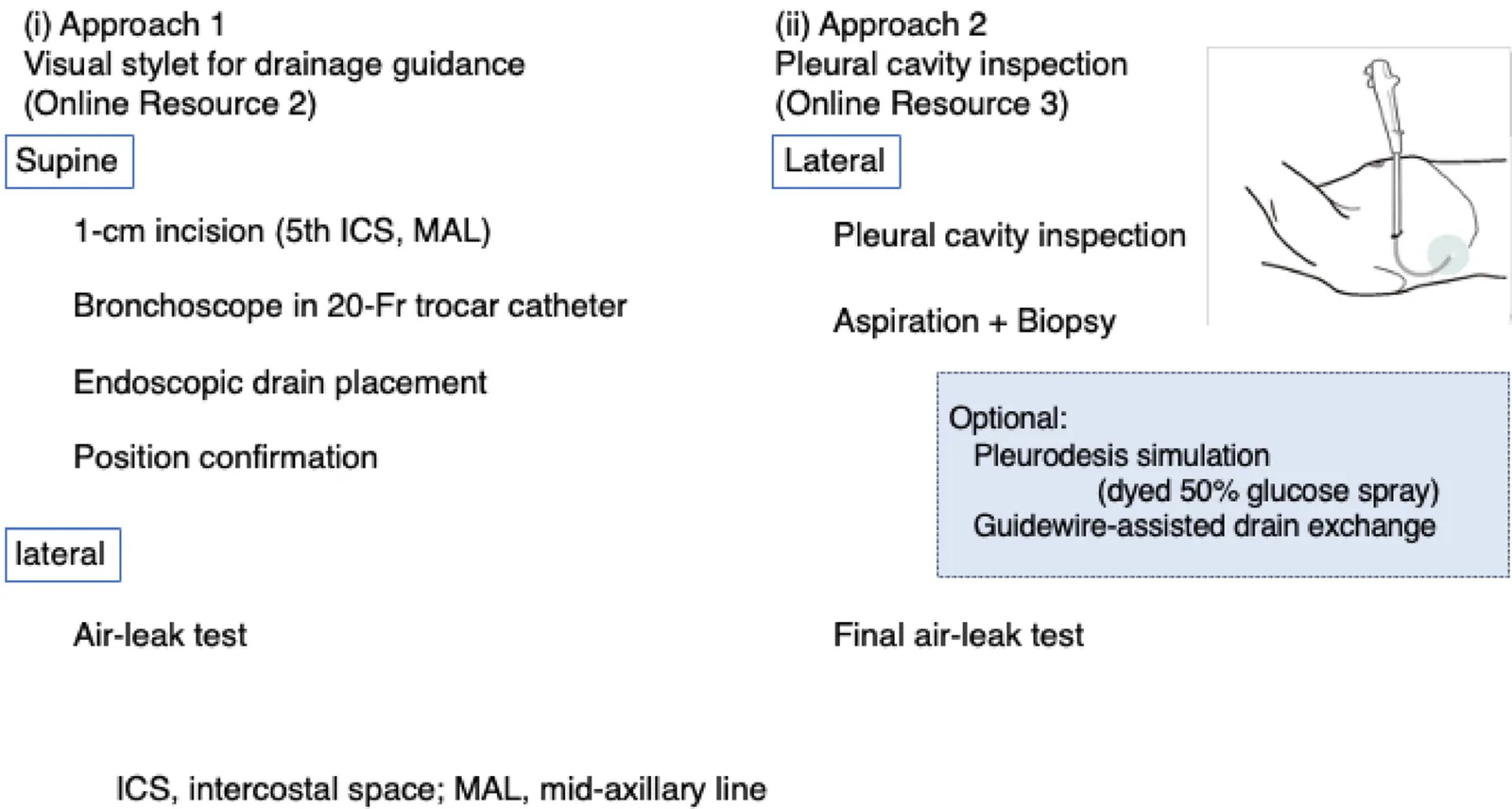
Procedural workflows: Approach 1 (visual stylet-guided drainage) and Approach 2 (pleural cavity inspection). ICS, intercostal space; MAL, mid-axillary line. The optional sprayed-instillation step is a maneuver simulation and does not imply pleurodesis efficacy

Three Thiel-embalmed cadavers (six hemithoraces) were intubated and ventilated. Pleural access was created via a 1 cm incision at the fifth intercostal space on the mid-axillary line. Approach 1 (visual stylet for drainage guidance) was assessed supine, and Approach 2 (pleural cavity inspection) was performed in the lateral decubitus position (Fig. 1).

The procedures were performed by five physicians: one trainee (8 years after graduation) and four dual-certified specialists (thoracic surgery and respiratory endoscopy). Procedure time was not systematically recorded; pleural viewing typically required several minutes. Thiel-embalmed cadavers may exhibit pleural adhesions and baseline air leaks, which can limit evaluability.

### Definitions of feasibility or success endpoints

The success endpoints of Approach 1 were: (i) visualization of pleural entry (pleural entry confirmed endoscopically) and (ii) direct-vision confirmation of intrapleural drain tip position.

Meanwhile, the endpoint of Approach 2 was the completion of a minimal diagnostic workflow comprising the following: (i) inspection of at least two pleural regions (apex, chest wall, and diaphragm); (ii) aspiration through the working channel; and (iii) direct-vision forceps pleural biopsy. An optional sprayed-instillation maneuver (simulation) was performed only to confirm technical deliverability and not to determine clinical efficacy. These workflows are illustrated in Fig. 1.

### Air-leak test (safety surrogate endpoint) and plateau definition

Safety was assessed after each approach using a standardized water-seal, air-leak test with a predefined plateau criterion (Online Resource 1). With the drain tip submerged in water, positive-pressure ventilation was applied and continuous bubbling was considered positive. A new persistent air leak was defined as negative-to-positive conversion between post-Approach 1 and post-Approach 2.

### Step-by-step technique

Approach 1: Visual stylet for drainage guidance (Online Resource 2).

A small-caliber single-use bronchoscope (2.7 mm outer diameter; 1.2 mm working channel) was loaded inside a 20-French trocar catheter and advanced under direct vision to confirm pleural entry. After visualization of intrapleural access, the drain was positioned and the bronchoscope withdrawn. The scope was reinserted as needed to reconfirm intrathoracic placement. Afterward, the air-leak test described above was performed.

Approach 2: Pleural cavity inspection (Online Resource 3).

Separately, the access tract was dilated, and a larger-caliber single-use bronchoscope (5.0 mm outer diameter; 2.2 mm working channel) was introduced as a flexible thoracoscope. The pleural cavity (apex, chest wall, and diaphragm) was inspected, focusing on maneuverability and stable close-to-surface viewing. Pleural fluid was aspirated via the working channel, and pleural biopsies were obtained using forceps under direct vision.

As an optional step, deliverability of a sprayed-instillation maneuver was simulated by spraying dyed 50% glucose at the apex; this was not intended to demonstrate pleurodesis distribution or efficacy. Adhesiolysis and definitive hemostasis were not attempted. When required, the drain was exchanged over a guidewire, followed by a repeat air-leak test.

### Outcomes in cadavers

Approach 1 achieved direct-vision pleural entry confirmation and chest drain placement or tip confirmation in 6/6 hemithoraces (Online Resource 4). The post-Approach 1 air-leak test was negative in 2/6 and positive in 4/6 hemithoraces.

Approach 2 was not attempted in specimen 2 (both hemithoraces) due to time constraints and a large continuous air leak after Approach 1; therefore, these hemithoraces were excluded from the feasibility assessment of Approach 2. In the four attempted hemithoraces (4/4), pleural viewing was assessed sequentially using aScope 4 Regular and aScope 5; pleural inspection (≥ 2 regions), aspiration, and direct-vision forceps pleural biopsy were feasible.

## Pitfalls, safety, and clinical positioning

### Clinical positioning and indications

These methods are adjuncts for rapid bedside drain confirmation (Approach1) or limited pleural biopsy (Approach2) when pleuroscopy or VATS resources or operating room access are unavailable. Compared with conventional blind tube insertion, they provide direct-vision pleural entry and tip confirmation. It is not intended for adhesiolysis, definitive hemostasis, or air-leak repair; in cases of dense adhesions, poor view, or bleeding, prompt abort-and-convert is required.

### Safety limitations and abort criteria

The working channel and hemostatic options are limited compared to standard pleuroscopy or VATS. The procedure should be aborted and converted to standard pleuroscopy or VATS (surgical management) if: (i) visualization cannot be maintained (persistent air leak, port leakage, or fogging/contamination); (ii) pleural adhesions prevent continuous cavity navigation; (iii) biopsy bleeding is not controllable with basic measures; or (iv) organ injury is suspected.

### Extrapolation to spontaneous breathing or local anesthesia

Compared with ventilated cadavers, spontaneous breathing adds respiratory, cardiac, or diaphragmatic motion that can impair visualization and handling. When introducing this technique into clinical practice, it is recommended to establish clear criteria for discontinuation, even though inserting a single-use bronchoscope into the thoracic cavity is within the scope of its intended indication.

### Cost considerations

Single-use systems add per-case scope costs but may reduce reprocessing burden and improve portability and infection-control logistics. For transparency, representative list prices in Japan for single-use bronchoscopes or monitor and an example acquisition cost of a reusable thoracoscopy system are provided in Online Resource 5. Actual costs vary by contract, case volume, and local reprocessing capacity.

## Conclusions

Single-use bronchoscopes enabled (i) direct-vision drain placement or tip confirmation using a fine-caliber scope as a visual stylet (Approach 1) and (ii) flexible pleural inspection with aspiration and direct-vision biopsy using a larger-caliber scope (Approach 2) in Thiel-embalmed cadavers. These approaches may serve as adjunct options in selected scenarios when standard pleuroscopy/thoracoscopy is not readily available, warranting stepwise clinical validation with strict indication and abort criteria.

## Supplementary Information

Below is the link to the electronic supplementary material.

Supplementary Material 1

Supplementary Material 2

Supplementary Material 3

Supplementary Material 4

Supplementary Material 5
