# Supplementary material for: Single-use bronchoscopes for pleural cavity access: cadaveric feasibility study
Source: Gen Thorac Cardiovasc Surg. 2026 May 11;74(9):913–6. doi: 10.1007/s11748-026-02309-4 (PMC13506559; doi:10.1007/s11748-026-02309-4)
Supplement: Supplementary file 1 [file 11748_2026_2309_MOESM1_ESM.docx]

Journal: General Thoracic and Cardiovascular Surgery
Article title: Single-Use Bronchoscopes for Pleural Cavity Access: Cadaveric Feasibility Study
Corresponding author: Yuki Sata (y.sata.0506@gmail.com)

**Online Resource 1. Air-leak test definition and plateau criteria (Text)**

Air-leak testing was performed at the end of Approach 1 and, when performed, at the end of Approach 2. The chest drain tip was submerged in a cup of water and positive-pressure ventilation was applied while observing bubbling.

Because transient bubbling may occur during initial evacuation of intrapleural gas (“space evacuation”), we defined a “plateau” as the time point at which bubbling decreases and ceases. Air-leak positivity was defined as continuous bubbling; negativity was defined as disappearance of bubbling after the plateau.

A new persistent air leak potentially attributable to pleural injury was defined as negative-to-positive conversion from the end of Approach 1 to the end of Approach 2. Hemithoraces already positive after Approach 1 were not evaluable for new-leak conversion.
