# Supplementary material for: Single-use bronchoscopes for pleural cavity access: cadaveric feasibility study
Source: Gen Thorac Cardiovasc Surg. 2026 May 11;74(9):913–6. doi: 10.1007/s11748-026-02309-4 (PMC13506559; doi:10.1007/s11748-026-02309-4)
Supplement: Supplementary file 2 [file 11748_2026_2309_MOESM4_ESM.docx]

Journal: General Thoracic and Cardiovascular Surgery
Article title: Single-Use Bronchoscopes for Pleural Cavity Access: Cadaveric Feasibility Study
Corresponding author: Yuki Sata (y.sata.0506@gmail.com)

**Online Resource 4. Summary of results (Table)**

**Table 1. Summary of results**

| No. | Side | Bronchoscope-guided drain placement (small-caliber) | Post-Approach 1 Air leak test | Thoracic viewing (aScope 4 Regular) | Thoracic viewing (aScope 5) | Post-Approach 2 Air leak test |
| --- | --- | --- | --- | --- | --- | --- |
| 1 | Right | Yes | Negative | Yes | Yes | Negative |
|  | Left | Yes | Positive | Yes | Yes | Positive |
| 2 | Right | Yes | Positive | - | - | - |
|  | Left | Yes | Positive | - | - | - |
| 3 | Right | Yes | Negative | Yes | Yes | Negative |
|  | Left | Yes | Positive | Yes | Yes | Positive |

Small-caliber bronchoscope: aScope 5 (outer diameter 2.7 mm; working channel 1.2 mm).

Large-caliber bronchoscopes for thoracic viewing: aScope 4 Regular (outer diameter 5.0 mm; working channel 2.2 mm) and aScope 5 (outer diameter 5.0 mm; working channel 2.2 mm).

Note: “–” indicates that Approach 2 and the post–Approach 2 air-leak test were not performed (time constraints and a large persistent air leak after Approach 1).

In hemithoraces in which Approach 2 was performed, pleural viewing was assessed sequentially using both aScope 4 Regular and aScope 5.
