# Supplementary material for: Single-use bronchoscopes for pleural cavity access: cadaveric feasibility study
Source: Gen Thorac Cardiovasc Surg. 2026 May 11;74(9):913–6. doi: 10.1007/s11748-026-02309-4 (PMC13506559; doi:10.1007/s11748-026-02309-4)
Supplement: Supplementary file 3 [file 11748_2026_2309_MOESM5_ESM.docx]

Journal: General Thoracic and Cardiovascular Surgery
Article title: Single-Use Bronchoscopes for Pleural Cavity Access: Cadaveric Feasibility Study
Corresponding author: Yuki Sata (y.sata.0506@gmail.com)

**Online Resource 5. Cost summary (Japan list prices; JPY)**

**Single-use bronchoscopes (sold in boxes of 5):**

- aScope 4 Broncho Regular: JPY 255,000 per box (JPY 51,000 per scope)

- aScope 5 Broncho 2.7/1.2 (fine-caliber): JPY 350,000 per box (JPY 70,000 per scope)

- aScope 5 Broncho 5.0/2.2 (HD model used for biopsy/scraping): JPY 475,000 per box (JPY 95,000 per scope)

**Monitor:**

- aView 2 Advance V2: JPY 900,000 per unit

**Example acquisition cost of a reusable thoracoscopy system:**

- Thoracoscope LTF-S190-10: JPY 4,000,000

- Monitor MLW-2124C: JPY 800,000

- Processor/light source OTV-S300: JPY 4,500,000

- Compact trolley TC-C3: JPY 270,000

- Installation fee: JPY 90,000

Total: JPY 9,660,000 (excluding recurring reprocessing cost and labor per use)

**Break-even point**

Even for the highest-cost model (Ambu aScope 5 Broncho 5.0/2.2), the break-even point was estimated at 92.2 procedures (approximately 93), or 101.7 procedures (approximately 102) if the monitor was already available; costs for reprocessing, repair, and personnel associated with reusable devices were not included.

Note: List prices may vary by institution/contract and may change over time.
